# An elevated monocyte-to-high-density lipoprotein–cholesterol ratio is associated with mortality in patients with coronary artery disease who have undergone PCI

**DOI:** 10.1042/BSR20201108

**Published:** 2020-08-17

**Authors:** Da-Peng Zhang, Gulinaer Baituola, Ting-Ting Wu, You Chen, Xian-Geng Hou, Yi Yang, Ying Pan, Xiang Ma, Ying-Ying Zheng

**Affiliations:** 1 Heart Center and Beijing Key Laboratory of Hypertension Disease, Beijing Chaoyang Hospital, Capital Medical University, Beijing, China; 2 Department of Cardiology, First Affiliated Hospital of Xinjiang Medical University, Urumqi 830011, P.R. China; 3 Department of Cardiology, First Affiliated Hospital of Zhengzhou University, Zhengzhou 450052, P.R. China

**Keywords:** Coronary artery disease, Monocyte to high-density lipoprotein cholesterol ratio, Mortality, Percutaneous coronary intervention

## Abstract

**Background:** The aim of the present study was to investigate the association between the monocyte-to-high-density lipoprotein–cholesterol ratio (MHR) and the outcomes of patients with coronary artery disease (CAD) who were treated with percutaneous coronary intervention (PCI).

**Methods:** A total of 5679 CAD patients from CORFCHD-PCI, a retrospective cohort study (identifier: ChiCTR-ORC-16010153), who underwent PCI were included in the study and divided into three tertiles according to their MHR values. The primary outcome was long-term mortality after PCI. The main secondary endpoints were stroke, readmission, and major adverse cardiovascular events (MACEs), defined as the combination of cardiac death, recurrent myocardial infarction, and target vessel reconstruction. The average follow-up time was 35.9 ± 22.6 months.

**Results:** Patients were divided into three groups according to MHR tertiles: the first tertile (MHR < 0.4; *n*=1290), second tertile (MHR ≥ 0.4–0.61; *n*=1878) and third tertile (MHR > 0.61; *n*=1870). The all-cause mortality (ACM) incidence was significantly lower in the first and second tertiles than in the third tertile (adjusted HR = 0.658, [95% CI: 0.408–0.903], *P*=0.009 and HR = 0.712, [95% CI: 0.538–0.941], *P*=0.017, respectively). Cardiac mortality (CM) occurred in 235 patients: 60 (3.1%) in the first tertile group, 74 (3.9%) in the second tertile group and 101 (5.4%) in the third tertile group. There was a significant difference in the CM incidence between the first tertile group and the third tertile group (HR = 0.581, [95% CI: 0.406–0.832], *P*=0.003), and there was also a difference in the CM incidence between the second tertile group and the third tertile group (HR = 0.690, [95% CI: 0.506–0.940], *P*=0.019).

**Conclusion:** The present study indicated that an increased MHR was independently associated with long-term mortality in CAD patients who have undergone PCI.

## Introduction

Coronary artery disease (CAD) is considered a complex disease, and the morbidity, mortality, disability, and recurrence rates are high [1,2]. CAD became one of the most common causes of death in the Chinese population in 2015 [3,4]. Atherosclerosis is the pathological basis of CAD. Arterial wall endothelial dysfunction and chronic inflammation continue to promote the development of atherosclerotic lesions [5]. Systemic inflammation, such as high levels of pro-inflammatory cytokines and adhesion molecules, actively promotes the transfer of monocytes to atherosclerotic lesions. Monocytes are important participants in the immune system [6]. The activation of inflammatory factors plays an important role in the initiation of atherosclerotic inflammation, which is involved in plaque progression, destabilization, rupture and thrombosis throughout the development of CAD. Hypercholesterolemia is one of the major risk factors for atherosclerosis. A large number of studies have shown that high-density lipoprotein (HDL) has protective effects against low-density lipoprotein (LDL) oxidation, transports cholesterol from the surrounding tissue to the liver for recycling, and inhibits the expression of endothelial adhesion molecules and the recruitment of monocytes in the arterial wall. HDL inhibits the inflammatory response by acting directly on monocytes. It has antioxidant properties and regulates vascular inflammation, vasomotor function, and thrombosis [7–9].

The monocyte-to-HDL-cholesterol ratio (MHR) has been proposed to have a role in systemic inflammation and to be a possible predictor of atherosclerosis development and progression. An increased MHR level was associated with adverse outcomes and was an indication of high rates of major cardiovascular adverse events (MACEs), including stent thrombosis and mortality after primary percutaneous coronary intervention (PCI) in ST-segment elevation myocardial infarction (STEMI) patients [10–13]. In addition, previous studies also suggested that the MHR is a useful predictor of outcomes in stable CAD patients or in acute coronary syndrome [14,15]. However, a few studies have evaluated the association of the MHR with long-term outcomes in Chinese patients with CAD who have undergone PCI. The aim of the present study was to investigate the association between the MHR and long-term outcomes in Chinese patients with CAD after PCI.

## Methods

### Study design and population

**Figure 1:**
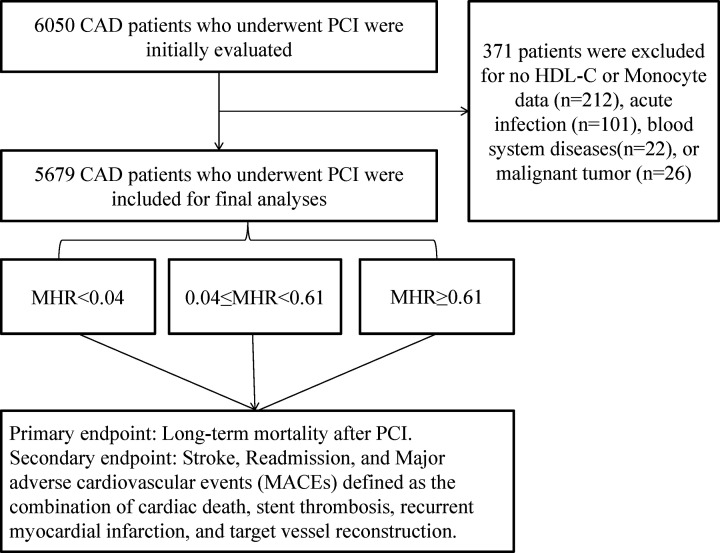
Flow chart of participant inclusion

From January 2008 to December 2016, a total of 5679 CAD patients who underwent PCI were enrolled, and all of them were from the CORFCHD-PCI study, which has been described in a previous article [16]. Briefly, CORFCHD-PCI is a large, single-center retrospective cohort study based on case records and a follow-up registry. The details of the design are registered at http://www.chictr.org.cn (identifier: ChiCTR-ORC-16010153). The PCIs were performed by experienced interventionalists. Initially, a total of 6050 patients with CAD who had undergone PCI were evaluated. A total of 371 patients were excluded due to no monocyte count or HDL-c data available; acute infections, including respiratory tract infections, urinary tract infections, oral infections, central nervous system infections, intestinal infections; blood system diseases; and malignant tumors. Finally, 5679 patients were enrolled in the present study. The flow chart for the inclusion and exclusion of the participants is shown in Figure 1.

### Blood testing

For the routine blood tests, 2-ml venous blood samples were collected in standardized dipotassium EDTA tubes. The routine blood samples were measured using an automated blood counter within 2 h of collection to minimize variations due to sample aging. We measured the serum concentrations of uric acid, total cholesterol, triglycerides, blood urea nitrogen (BUN), creatinine (Cr), low-density lipoprotein (LDL), high-density lipoprotein (HDL), and fasting glucose using chemical analysis equipment (Dimension AR/AVL Clinical Chemistry System, Newark, NJ) in the Clinical Laboratory Department of the First Affiliated Hospital of Xinjiang Medical University, as described previously [17].

### Calculation of the MHR

We calculated the MHR by dividing the absolute value of the monocyte count by that of the HDL-C. The normal range of monocytes is (0.12–0.8) × 10^9^/l. The normal range of HDL-C is 0.78–2 mmol/l.

### Clinical and demographic characteristics collection

We collected demographic data, laboratory data, including routine blood parameters and biochemical indicators, cardiovascular risk factor data, including smoking status, alcohol consumption, previously diagnosed diabetes, history of hypertension, familial history of CAD and history of medication and surgical disease, ECG data, data from echocardiography, coronary angiography and PCI procedures, and the short-term and long-term outcomes of these patients. The medical history included the use of antiplatelet therapy, CCBs, ACEIs or ARBs, β-blockers, and statins.

According to the diagnostic criteria for hypertension, a patient was considered to have hypertension if he was undergoing active treatment with antihypertensive drugs or had a blood pressure ≥140/90 mmHg on at least two separate occasions [18]. A diagnosis of diabetes mellitus was considered positive in patients with a definite history of diabetes who were undergoing treatment with glucose-lowering agents, in those with a fasting glucose level of at least 7.1 mmol/l, and in those with a 2-h postload glucose level of at least 11.1 mmol/l [19]. The diagnosis of hyperlipidemia comes from the ‘guidelines for the prevention and treatment of dyslipidemia in Chinese adults (2016)’ [20]. The smoking status classifications were current smokers, former smokers, and never-smokers. Persons reporting regular tobacco use in the previous 6 months were considered current smokers.

### Ethical approval of the study protocol

The study protocol was consistent with the Declaration of Helsinki and approved by the Institutional Ethics Committee of the First Affiliated Hospital of Xinjiang Medical University.

### Endpoints

The primary endpoints were long-term all-cause mortality (ACM) and cardiac mortality (CM). Deaths were considered to be a result of a cardiac condition unless a definite noncardiac cause of death was identified. Secondary endpoints included stroke, bleeding events, readmission, and major adverse cardiac events (MACEs). MACEs were defined as the combination of cardiac death, recurrent myocardial infarction and target vessel reconstruction.

### Follow-up

All patients were scheduled for elective clinical follow-up at 1 month, 3 months, 6 months, 1 year, 3 years, and 5 years. We clinically monitored the patients for cardiovascular events and their medication status. The patients were followed up for at least 2 years, and the longest follow-up time was 10 years. The investigators followed the patients by either office visits or telephone contact as necessary.

### Statistical analysis

Data were analyzed by using SPSS 22.0 software. The continuous variables are presented as the mean ± standard deviation (SD), and the categorical variables are presented as the number of patients and percentages. The MHR tertiles categorized into three groups by trisection (<0.04, ≥0.4 U/l to 0.61 and >0.61) and compared with one-way ANOVA (for continuous variables). Kruskal–Wallis tests (for nonparametric variables) and chi-square tests or Fisher’s exact tests were performed for the categorical variables. The chi-square test was employed for the comparison of categorical variables. Kaplan–Meier analysis was applied to the cumulative incidence rates of the long-term outcomes, and the log-rank test was used to compare groups. Multivariable analysis was performed to assess the predictive value of the MHR for outcomes during and up to a 10-year follow-up. Hazard ratios (HRs) and 95% confidence intervals (CIs) were calculated. A *P*<0.05 (two-sided) was considered significant.

## Results

### Baseline data

The study included 5679 patients who were divided into three groups according to MHR: the first tertile (MHR < 0.04; *n*=1923), second tertile (MHR ≥ 0.04–0.61; *n*=1880), and third tertile (MHR ≥ 0.61; *n*=1876). The baseline data are shown in Table 1. All patients were administered dual antiplatelet therapy with aspirin and one P2Y12 receptor antagonist after PCI. Several variables were significantly different among these three tertiles, including age, sex, smoking status, blood urea nitrogen (BUN), creatinine (Cr), uric acid (UA), triglycerides (TG), total cholesterol (TC), HDL, LDL, and Apo-a1 (all *P*<0.05). There was no significant difference in hypertension, systolic blood pressure (SBP), diastolic blood pressure (DBP), diabetes mellitus, hyperlipidemia, blood glucose, Apo-B, heart failure, or stroke.

**Table 1:** Baseline characteristics of patients

Variables	MHR			*P*
	Tertile 1 (*n*=1923)	Tertile 2 (*n*=1880)	Tertile 3 (*n*=1876)	
Age (years)	60.41 ± 10.34	59.64 ± 10.78	58.36 ± 11.14	<0.001
Gender (male)	1277 (66.4%)	1422 (75.6%)	1523 (81.2%)	<0.001
Smoking	637 (35%)	756 (40.2%)	852 (45.4%)	<0.001
Hypertension	818 (42.5%)	793 (42.2%)	814 (43.4%)	0.743
SBP	127.65 ± 18.98	127.13 ± 18.93	126.42 ± 18.35	0.126
DBP	76.28 ± 11.30	76.05 ± 11.25	76.57 ± 11.41	0.381
Diabetes mellitus	455 (23.7%)	481 (25.6%)	452 (24.1%)	0.352
Hyperlipidemia	163 (8.5%)	162 (8.7%)	174 (9.3%)	0.647
BUN	5.43 ± 1.58	5.51 ± 1.66	5.62 ± 1.76	0.003
Cr	73.25 ± 19.36	76.29 ± 20.58	78.43 ± 20.02	<0.001
UA	315.18 ± 86.55	325.95 ± 89.60	328.61 ± 94.12	<0.001
Blood glucose	6.59 ± 3.21	6.57 ±3.17	6.70 ± 3.90	0.453
TG	1.73 ± 1.19	1.90 ± 1.21	2.07 ± 1.37	<0.001
TC	4.09 ± 1.13	3.98 ± 1.10	3.80 ± 107	<0.001
HDL	1.25 ± 0.71	0.98 ± 0.21	0.82 ± 0.21	<0.001
LDL	2.55 ± 0.95	2.47 ± 0.89	2.36 ± 0.89	<0.001
Apo-A1	1.26 ± 0.35	1.16 ± 0.28	1.08 ± 0.29	<0.001
Apo-B	0.86 ± 0.39	0.85 ± 0.39	0.84 ± 0.41	0.22
Lp(a)	219.65 ± 177.99	215.62 ± 168.88	225.67 ± 183.16	0.215
CCB (*n*,%)	226 (11.8)	210 (11.2)	207(11.1)	0.734
β-Blockers (*n*,%)	799 (41.8)	727 (38.8)	775 (41.5)	0.125
ARB (*n*,%)	448 (23.5)	433(23.2)	410(21.9)	0.488
Statin (*n*,%)	1072 (56.3)	992 (53.2)	984(52.9)	0.065

Abbreviations: ApoA1, apolipoprotein A1; ApoB, apolipoprotein B; BUN, blood urea nitrogen; Cr, creatinine; DBP, diastolic blood pressure; HDL-C, high-density lipoprotein cholesterol; Lp(a), lipoprotein a; LDL-C, low-density lipoprotein cholesterol; SBP, systolic blood pressure; TC, total cholesterol; TG, triglyceride; UA, uric acid.

### Clinical outcome

**Figure 2:**
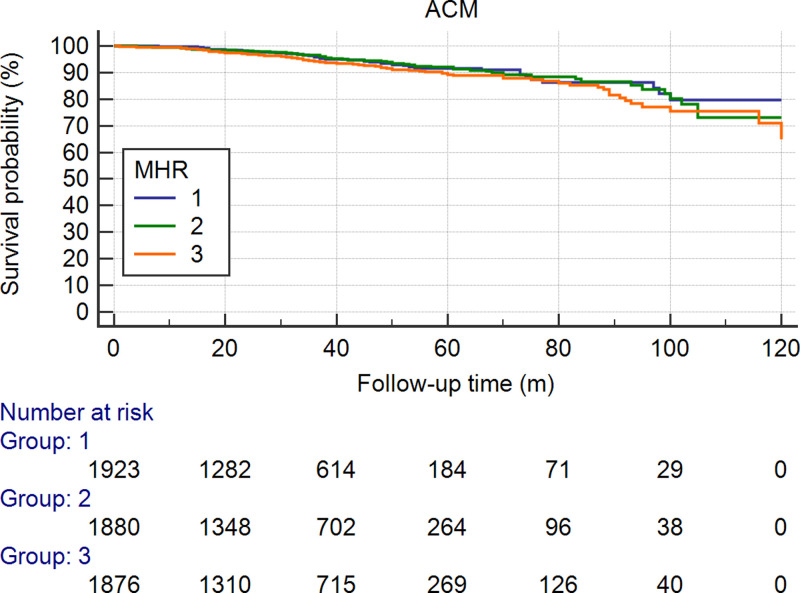
Cumulative Kaplan–Meier estimates of the time to the first adjudicated occurrence of ACM

**Figure 3:**
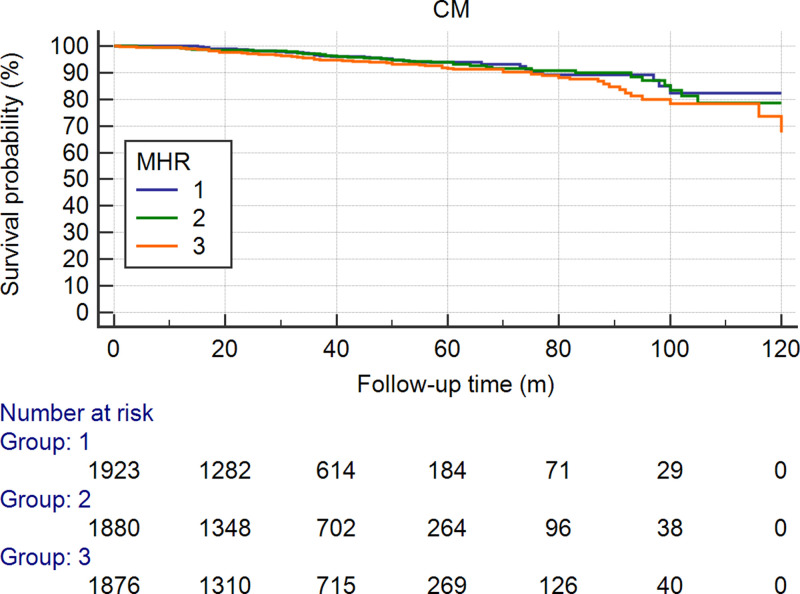
Cumulative Kaplan–Meier estimates of the time to the first adjudicated occurrence of CM

**Figure 4:**
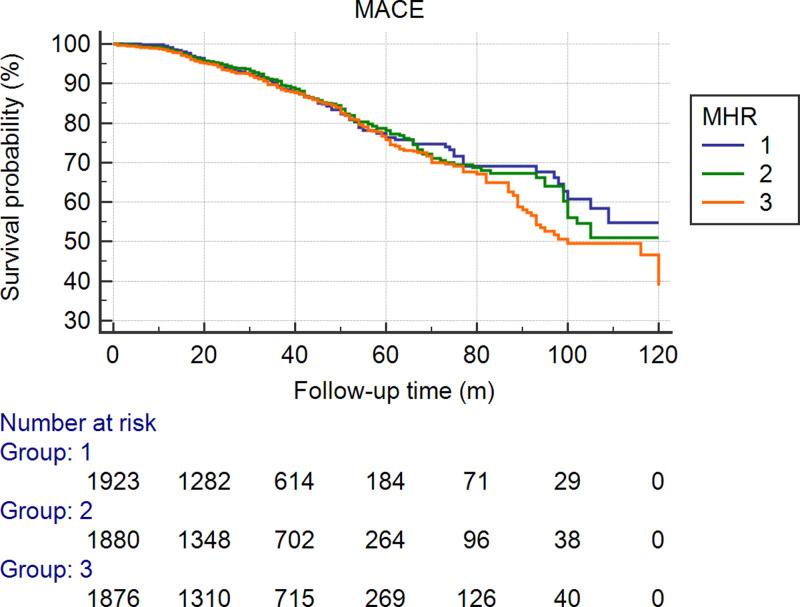
Cumulative Kaplan–Meier estimates of the time to the first adjudicated occurrence of a MACE

To predict mortality, the MHR (AUC = 0.600, 95% CI: 0.529–0.670, *P*=0.005) was stronger than monocytes (AUC = 0.532, 95% CI: 0.465–0.600, *P*=0.363) or HDL-C (AUC = 0.513, 95% CI: 0.442–0.583, *P*=0.722) (Supplementary Tables S1 and S2). There were 293 (5.16%) ACMs during the follow-up. The incidence of ACM in the first tertile was 80 (4.2%), it was 91 (4.8%) in the second tertile, and it was 122 (6.5%) in the third tertile. The ACM incidence was significantly lower in the first tertile than in the second tertile or in the third tertile (both *P*<0.05). The ACM incidence was significantly lower in the first and second tertiles than in the third tertile (adjusted HR = 0.658 [0.408–0.903], *P*=0.009 and HR = 0.712 [0.538–0.941], *P*=0.017, respectively). As shown in Figure 2, Tables 2 and 3, the incidence of CM in the first tertile was 60 (3.1%), which was lower than that in the second tertile [74 (3.9%)] and third tertile [101 (5.4%)]. Univariate Cox regression analysis also showed significant differences in CM between patients in the third tertile and those in the first tertile (HR = 0.581, 95% CI: 0.406–0.832, *P*=0.003) and second tertile (HR = 0.690, 95% CI: 0.506–0.9401, *P*=0.019). As shown in Figure 3, Tables 2 and 4, we also found that the incidence of MACEs was higher in the third tertile group than in the first tertile group (*P*=0.002) in a univariate analysis. However, the difference was not significant after multivariable Cox regression analysis, as shown in Figure 4, Tables 2 and 5. Since the MHR was a continuous variable, we also performed a restricted cubic spline regression to show the relation between the MHR and the hazard risk. The results are shown in Supplementary Figure S1.

**Table 2:** Clinical outcomes among the three groups

Variables	MHR			*P*
	Tertile 1 (*n*=1923)	Tertile 2 (*n*=1880)	Tertile 3 (*n*=1876)	
ACM	80 (4.2%)	91 (4.8%)	122 (6.5%)	0.004
CM	60 (3.1%)	74 (3.9%)	101 (5.4%)	0.002
MACE	258 (13.4%)	276 (14.7%)	327 (17.4%)	0.002
HF	49 (2.5%)	59 (3.1%)	60 (3.2%)	0.424
Stroke	24 (1.2%)	21 (1.1%)	30 (1.6%)	0.408
Bleeding	52 (2.7%)	62 (3.3%)	52 (2.8%)	0.495
Readmission	263(13.7%)	247(13.1%)	265(14.1%)	0.677
Secondary MI	56(2.9%)	66(3.5%)	59(3.1%)	0.571
Secondary PCI	80 (4.2%)	83 (4.4%)	89 (4.7%)	0.681
Secondary CABG	20 (1.0%)	15 (0.8%)	15 (0.8%)	0.654
TVR	99 (5.1%)	96 (5.1)	103 (5.5)	0.845

Abbreviations: ACM, all-cause mortality; CABG, coronary artery bypass grafting; CM, cardiac mortality; MACE, major adverse cardiovascular events; hF, heart failure; MI, myocardial infarction; PCI, percutaneous coronary intervention.

**Table 3:** Multivariable Cox regression analysis of ACM

Variables	*B*	*SE*	*Wald*	*P*	*HR*	*95%CI*
Gender (male)	-0.056	0.153	0.134	0.714	0.945	0.701–1.276
Age (years)	0.028	0.006	21.655	0	1.028	1.016–1.040
Smoking	0.035	0.135	0.066	0.797	1.035	0.795–1.348
BUN	0.055	0.036	2.281	0.131	1.057	0.984–1.135
Cr	0.004	0.003	1.898	0.168	1.004	0.998–1.009
TG	-0.022	0.049	0.206	0.65	0.978	0.889–1.076
TC	0.117	0.088	1.786	0.181	1.124	0.947–1.335
HDL	0.155	0.112	1.903	0.168	1.168	0.937–1.455
LDL	-0.184	0.105	3.096	0.078	0.832	0.677–1.021
Apo-AI	0.047	0.198	0.058	0.81	1.049	0.712–1.545
UA	0	0.001	0.06	0.806	1	0.999–1.002
MHR classify			8.601	0.014		
MHR(1)	-0.418	0.161	6.726	0.009	0.658	0.480–0.903
MHR(2)	-0.34	0.142	5.69	0.017	0.712	0.538-0.941

**Table 4:** Multivariable Cox regression analysis of CM

Variables	*B*	*SE*	*Wald*	*P*	*HR*	95%CI
Gender (male)	−0.064	0.171	0.139	0.71	0.938	0.671–1.312
Age [years]	0.019	0.007	8.174	0.004	1.019	1.006–1.032
Smoking	0.131	0.151	0.757	0.384	1.14	0.848–1.533
BUN	0.08	0.04	3.968	0.046	1.083	1.001–1.172
Cr	0.005	0.003	2.432	0.119	1.005	0.999–1.010
TG	−0.079	0.058	1.844	0.175	0.924	0.824–1.036
TC	0.192	0.093	4.28	0.039	1.212	1.010–1.454
HDL	0.158	0.131	1.447	0.229	1.171	0.905–1.516
LDL	−0.225	0.112	4.008	0.045	0.799	0.641–0.995
Apo-AI	0.068	0.218	0.098	0.754	1.071	0.698–1.643
UA	0	0.001	0.007	0.932	1	0.999–1.002
MHR classify			10.171	0.006		
MHR(1)	−0.543	0.183	8.786	0.003	0.581	0.406–0.832
MHR(2)	−0.371	0.158	5.539	0.019	0.69	0.506–0.9401

**Table 5:** Multivariable Cox regression analysis of MACE

Variables	*B*	SE	Wald	*P*	HR	95%CI
Gender (male)	0.098	0.092	1.138	0.286	1.103	0.921–1.320
Age [years]	0.004	0.003	1.672	0.196	1.004	0.998–1.011
Smoking	0.199	0.078	6.515	0.011	1.22	1.047–1.421
BUN	0.049	0.022	4.979	0.026	1.05	1.006–1.096
Cr	0	0.002	0	0.988	1	0.996–1.004
TG	-0.014	0.03	0.213	0.645	0.986	0.931–1.045
TC	0.035	0.056	0.389	0.533	1.035	0.928–1.155
HDL	0.022	0.084	0.066	0.797	1.022	0.867–1.204
LDL	-0.11	0.066	2.807	0.094	0.896	0.787–1.019
Apo-AI	-0.133	0.126	1.125	0.289	0.875	0.684–1.120
UA	0.001	0	1.55	0.213	1.001	1.000–1.001
MHR classify			3.199	0.202		
MHR(1)	-0.089	0.094	0.903	0.342	0.915	0.762–1.099
MHR(2)	-0.15	0.084	3.191	0.074	0.861	0.730–1.015

## Discussion

To the best of our knowledge, a few studies have evaluated the association of the MHR with long-term outcomes in Chinese CAD patients who have undergone PCI. The present study indicated that an increased MHR was independently associated with long-term mortality in CAD patients who underwent PCI in China. A higher MHR was one of the strongest independent predictors of long-term mortality. The patients with CAD who underwent PCI and were in the highest MHR quartile had the highest mortality risk.

The inflammatory response has a vital function in the progression of atherosclerosis. Bath et al. [21] reported that monocytes in patients with hypercholesterolemia were more sensitive to the stimulation of chemokines. This may explain the increased involvement of monocytes in the formation of atherosclerosis associated with high cholesterol. Murphy et al. [22] reported that HDL-C has anti-inflammatory effects on human monocytes by inhibiting the activation of CD11b. HDL-C inhibits LDL-C oxidation by reducing monocyte chemotaxis and monocyte chemotaxis protein 1 expression, thereby affecting the inflammatory index. The MHR is a novel inflammation-based marker and may be an independent predictor of future cardiovascular events [23]. Previous studies have shown that a higher MHR is associated with thrombus burden and mortality in STEMI patients who undergo successful primary PCI [10–13]. A higher MHR can predict CIN development after primary PCI in STEMI patients [24], and it is also a powerful predictor of in-stent restenosis in patients with stable or unstable angina pectoris who have undergone successful bare metal stent implantation [25]. Sercelik et al. [26] found that the MHR was significantly higher in the STEMI group than in the control group and in the high TIMI score group than in the low TIMI score group.

In the past two decades, China’s rapid economic growth has led to dramatic changes in people’s lifestyles, transportation patterns and eating habits. The number of deaths due to CAD has doubled, reaching 1 million per year [27]. Although the development of CAD is unclear, its etiology and interaction with lifestyle are undoubtedly complex. Inflammation and lipid accumulation are two basic hallmarks of CAD [28]. A high monocyte count and low HDL-C levels may be relevant to inflammation and oxidative stress [29–33]. It has been reported that the MHR is a new prognostic marker for several CVDs [32,34].

In the present study, we included 5679 CAD patients to investigate the relationship between the MHR and outcomes and found that the MHR is an independent predictor after adjustment for confounders. Therefore, the MHR can be used as a valuable and low-cost predictor of clinical outcomes in CAD patients who have undergone PCI. Furthermore, this predictor is worthy of application in clinical practice. The large sample size is a strength of our study. However, there were also several limitations that should be mentioned. We only collected baseline data for monocytes and HDL-C in the present study. The single retrospective cohort design is another limitation.

## Conclusion

In conclusion, the present study suggests that the baseline MHR is a simple, inexpensive and independent predictor of mortality in CAD patients who have undergone PCI. An elevated MHR was associated with all-cause mortality and cardiac mortality in CAD patients.

## Supplementary Material

Supplementary Figure S1 and Tables S1-S2

## Abbreviations

CAD: coronary artery disease
HDL: high-density lipoprotein
LDL: low-density lipoprotein
MACE: major cardiovascular adverse event
MHR: monocyte-to-high-density lipoprotein–cholesterol ratio
PCI: percutaneous coronary intervention
