# Supplementary material for: An elevated monocyte-to-high-density lipoprotein–cholesterol ratio is associated with mortality in patients with coronary artery disease who have undergone PCI
Source: Biosci Rep. 2020 Aug 17;40(8):BSR20201108. doi: 10.1042/BSR20201108 (PMC7432996; doi:10.1042/BSR20201108)
Supplement: Supplementary Figure S1 and Tables S1-S2 [file BSR-2020-1108_supp.pdf]

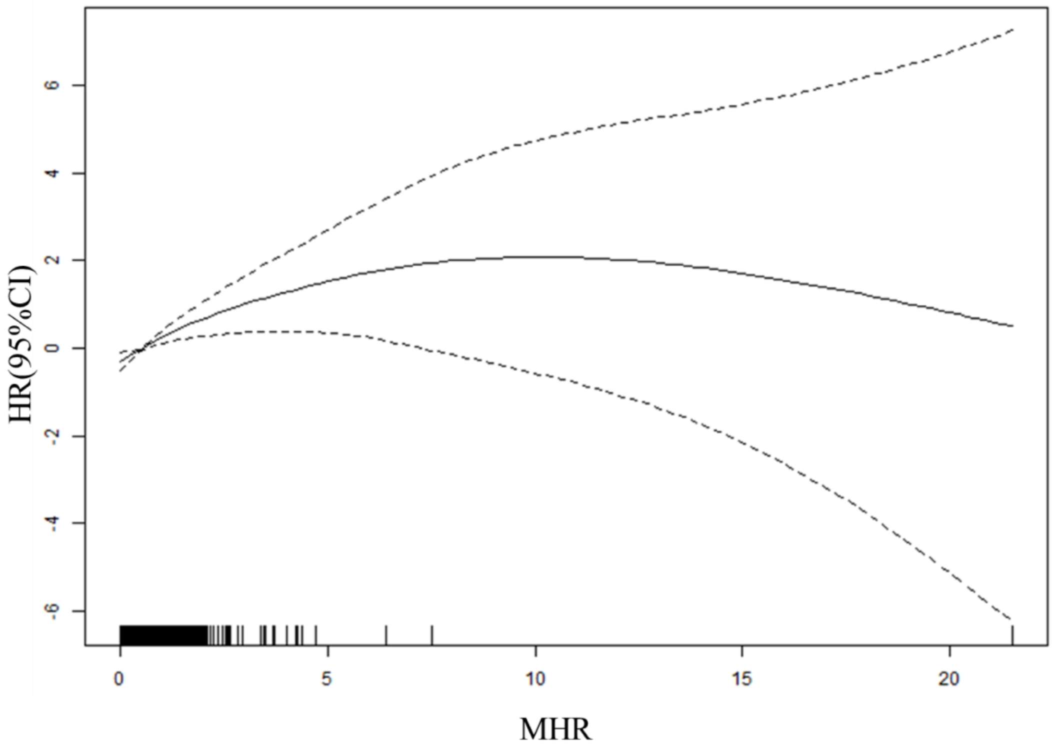

Figure S1. The restricted cubic spline regression result.

Table S1. Comparison of the Area Under the Curve among three variables.

Test Result Variable(s)	Area	Std. Error[a]	Asymptotic Sig.[b]	Asymptotic 95% Confidence Interval	
				Lower Bound	Upper Bound
Monocyte	0.532	0.034	0.363	0.465	0.600
HDL	0.513	0.036	0.722	0.442	0.583
MHR	0.600	0.036	0.005	0.529	0.670

Area Under the Curve

Test Result Variable(s)	Area	Std. Error[a]	Asymptotic Sig.[b]	Asymptotic 95% Confidence Interval	
				Lower Bound	Upper Bound
age	0.576	0.019	0.000	0.539	0.613
smoking	0.515	0.019	0.447	0.448	0.523
drinking	0.512	0.019	0.541	0.451	0.526
diabetes	0.509	0.019	0.648	0.471	0.547
hypertension	0.503	0.019	0.868	0.465	0.541
**MHR**	**0.600**	**0.036**	**0.005**	**0.529**	**0.670**
BUN	0.549	0.020	0.011	0.509	0.589
Cr	0.563	0.020	0.001	0.522	0.603
UA	0.507	0.019	0.710	0.469	0.545
TG	0.509	0.019	0.624	0.454	0.527
TC	0.513	0.020	0.488	0.475	0.552
HDL	0.511	0.019	0.586	0.452	0.527
LDL	0.504	0.020	0.851	0.465	0.542
